# From Thirst to Satiety: The Anterior Mid-Cingulate Cortex and Right Posterior Insula Indicate Dynamic Changes in Incentive Value

**DOI:** 10.3389/fnhum.2017.00234

**Published:** 2017-05-11

**Authors:** Christoph A. Becker, Tobias Flaisch, Britta Renner, Harald T. Schupp

**Affiliations:** Department of Psychology, University of KonstanzKonstanz, Germany

**Keywords:** deprivation, drinking, fMRI, motivation, moment-to-moment

## Abstract

The cingulate cortex and insula are among the neural structures whose activations have been modulated in functional imaging studies examining discrete states of thirst and drinking to satiation. Building upon these findings, the present study aimed to identify neural structures that change their pattern of activation elicited by water held in the mouth in relation to the internal body state, i.e., proportional to continuous water consumption. Accordingly, participants in a thirsty state were scanned while receiving increments of water until satiety was reached. As expected, fluid ingestion led to a clear decrease in self-reported thirst and the pleasantness ratings of the water ingested. Furthermore, linear decreases in the blood oxygenation level dependent (BOLD) response to water ingestion were observed in the anterior mid-cingulate cortex (aMCC) and right posterior insula as participants shifted towards the non-thirsty state. In addition, regions in the superior temporal gyrus (STG), supplementary motor area (SMA), superior parietal lobule (SPL), precuneus and calcarine sulcus also showed a linear decrease with increasing fluid consumption. Further analyses related single trial BOLD responses of associated regions to trial-by-trial ratings of thirst and pleasantness. Overall, the aMCC and posterior insula may be key sites of a neural network representing the motivation for drinking based on the dynamic integration of internal state and external stimuli.

## Introduction

Keeping fluid levels in balance is crucial for survival (Rolls and Rolls, 1982). Accordingly, neural systems evolved regulating the motivation to initiate and terminate fluid ingestion. Everybody knows that even short periods without water can lead to the sensation of thirst and the desire to drink. However, over the course of drinking, pleasure and incentive motivation for drinking decreases rapidly, resulting in the termination of drinking. Accordingly, continuous consumption of water can serve as a model system for identifying neural structures changing their pattern of activation to water in relation to the internal body state.

Behavioral studies have systematically explored the effects of dehydration on drinking and fluid restoration. Specifically, gradual dehydration of intracellular and extracellular body fluid compartments is proportionally linked with increases in water intake in rodents, dogs and monkeys (Rolls and Rolls, 1982). Furthermore, neurophysiological studies have investigated the sensing of dehydration using structures such as the hypothalamus and circumventricular organs (Johnson and Thunhorst, 1997; Bourque, 2008). Tracing studies in rodents further revealed that these osmoregulatory signals are projected via thalamocortical pathways to the insula and the cingulate cortex (Hollis et al., 2008; Farrell et al., 2011), providing a pathway to need-dependent adjustments of stimulus value and behavior.

In humans, neural correlates of thirst have mainly been investigated by comparing pharmacologically induced states of dehydration with satiety. In a positron emission tomography (PET) study, Denton et al. (1999) gave participants NaCl infusions to increase blood osmolarity, which led to strong feelings of thirst. A positive association between Na plasma level and brain activation was observed in the anterior cingulate, posterior parietal, middle temporal and superior temporal cortices, accompanied by deactivations in the inferior, middle and superior frontal cortices. Further, PET and functional magnetic resonance (fMRI) studies have corroborated these findings, reporting reliable activations in deprived state as well as post-satiety deactivations in anterior-, mid-cingulate (ACC, MCC) and insular cortices (Denton et al., 1999; Parsons et al., 2000; Egan et al., 2003; Farrell et al., 2006, 2008, 2011). In addition, a positive relationship between thirst experience and brain activity was observed in posterior and anterior regions of the cingulate cortex (Denton et al., 1999; Farrell et al., 2008). Collectively, these studies suggest that dehydration provides a powerful stimulus that activates the motivation for drinking associated with widespread shifts in patterns of activation and deactivation of resting-state brain activity.

The motivation for drinking rests on the dynamic integration of internal and external stimuli (Toates, 1986). A host of cues associated with mouth sensation, swallowing, and gastro-intestinal sensation provides the basis for terminating drinking well before fluid balance is restored (Rolls, 2005). Thus, drinking provides a model system for studying motivation processes that translate moment-to-moment variations of internal and external stimuli into a state value that regulates physiological, cognitive, and behavioral responding (Morrison and Salzman, 2010). A previous study by de Araujo et al. (2003) examined neural activity to drinking water when participants were in either a deprived or satiated state. While the activations elicited by ingesting water were not modulated by body state in some regions of the brain, i.e., cortical taste areas, other regions were only responsive when in a thirst state, i.e., middle insula and caudal orbitofrontal cortex. Furthermore, regions within the ACC, middle insula, and orbitofrontal cortex showed a positive relationship to self-reported pleasantness (de Araujo et al., 2003; see also Saker et al., 2014). These findings demonstrate neural regions critical for integrating internal body state with need-related external stimuli. However, previous studies compared discrete body states of dehydration and satiety, leaving open the issue of neural correlates of the dynamic moment-to-moment regulation of incentive value of water.

The main goal of the present study was to examine whether the activation in neural structures implicated in the regulation of drinking is proportional, on a moment-to-moment basis, to the amount of water consumed and the associated decrease of incentive value of water. Accordingly, participants were mildly deprived (~7 h of fluid deprivation) and repeatedly received (50 times) small sips of water (10 ml), sufficient to satisfy their thirst. According to previous findings, the main hypothesis was that regional brain activity in the ACC, MCC and insular cortex elicited by water linearly decreases with increasing amount of fluid ingested. This hypothesis was examined by comparing blood oxygenation level dependent (BOLD) signal activity to identical water stimuli as a function of stimulus repetition, i.e., five time bins based on 10 trials each. Furthermore, a follow-up analysis determined the correlation between single trial BOLD responses and ratings of thirst as well as pleasure given for each trial for the neural regions indicated by the main analysis.

## Materials and Methods

### Participants

Twenty-four participants (12 females, 6 left-handed) between 19 and 27 years of age (*M* = 21.1) with normal or corrected-to-normal vision participated in the study. Participants were recruited at the University of  Konstanz and received either course credit or €8 per hour. The study was approved by the ethics committee of the University of Konstanz, and written informed consent was acquired from all participants. Participants were not included in the study if they had a history or currently suffered from psychiatric, neurological, or endocrine diseases or were taking medication that affects the endocrine or central nervous system.

### Stimulus Material

Behavioral studies suggest that people drink between 200–600 ml of water after a period of fluid depletion (Rolls et al., 1980; Boulze et al., 1983; Phillips et al., 1984). Accordingly, the stimulus material comprised 530 ml mineral water (Evian, France), split into 30 ml for familiarization with the water delivery and 500 ml for the main experiment. The water was delivered at room temperature. The administration of small amounts (10 ml) was realized with an electronically controlled syringe connected to a plastic tube and a mouthpiece. The plastic tube was attached to the MRI coil so that the mouthpiece ended behind the participants’ lips and water could easily flow into the mouth.

### Procedure

An initial screening session served to inform participants about the study, including the requirement to refrain from drinking to evoke thirst, and check their eligibility for fMRI scanning. To control for variations in circadian rhythm, all sessions were scheduled at 6 pm. Participants had to completely refrain from drinking for 7 h.

**Figure 1:**
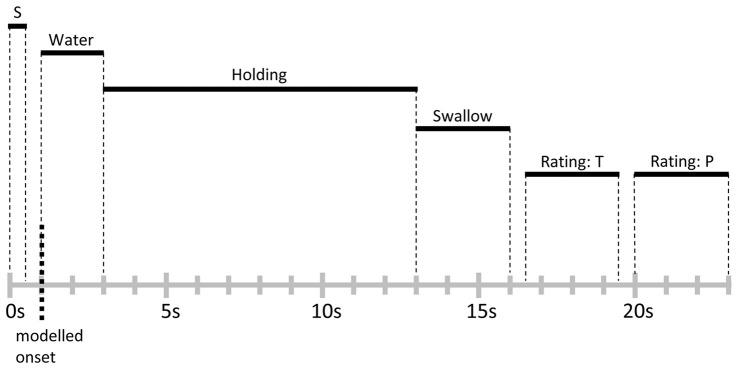
**Schematic representation and timing of a trial**. Each trial lasted for 23 s and was followed by a variable inter-trial interval (ITI) of 2–5 s. Abbreviations: S, signal cue; T, thirst rating; P, stimulus pleasantness rating.

As a manipulation check, participants provided ratings of thirst at the beginning of the functional imaging session. All participants indicated high levels of thirst and reported that they had complied with the deprivation instruction. Furthermore, to probe level of food deprivation, participants were asked to provide hunger ratings, and changes across time were assessed by collecting ratings immediately after the functional imaging condition and the end of the experiment. Inside the scanner, participants were familiarized with the trial structure by performing three trials of water delivery without MRI acquisition. None of the participants reported any discomfort or ambiguity with the task. Furthermore, participants were asked to keep body and head movements at minimum and to closely follow the timing of the trials described below. The main experiment consisted of 50 trials. As illustrated in Figure 1, each trial started with the word “water” presented for 0.5 s onto a projection screen positioned in front of the participant’s eyes, which signaled subsequent water delivery. Following a 0.5 s inter-stimulus interval (ISI), 10 ml of water were administered to the participant with a rate of 5 ml/s, i.e., 2 s water delivery period. Next, the label “hold” was presented for 10 s, indicating the participants should hold the water in their mouths without swallowing. Then, the word “swallow” was presented for 3 s, signaling the participant to swallow the water during this time period. Afterwards, separated by 0.5 s ISIs, ratings of thirst and stimulus pleasantness were collected during two 3 s time periods. Each trial was followed by a variable inter-trial interval (ITI), where a white fixation cross was displayed on a black background. The ITI was exponentially distributed with a mean of 3 s and a range of 2–5 s (see for example Amaro and Barker, 2006). At the end of the experimental task, a T1-weighted structural scan was recorded and participants debriefed.

### Self-Report Data

Participants provided ratings on the subjective experience of thirst and the pleasure associated with water consumption for each trial on a 7-point Likert scale ranging from 1 (not thirsty/not pleasant) to 7 (very thirsty/very pleasant). The ratings were obtained with a two-button, fiber-optic response box (Lumina LS-Pairs, Cedrus Corporation^1^) which was attached to the left or right hand, balanced across participants. Each rating started with a graphically highlighted cursor located at the midpoint of the rating scale. Each button press moved the cursor stepwise towards the left or right anchor, respectively. The selected ratings were recorded after the 3 s rating periods. Practice trials familiarized the participant with the rating procedure. To correspond to the main functional imaging data analysis, thirst and pleasure ratings were merged into five time bins (trial 01–10, trial 11–20, trial 21–30, trial 31–40, trial 41–50) and entered into a repeated measures analyses of variance (ANOVA) with the factor *Time* (Bin1 vs. Bin2 vs. Bin3 vs. Bin4 vs. Bin5).

Furthermore, before and after the functional imaging session and at the end of the experimental session, participants provided hunger and thirst ratings on a 7-point Likert scale, ranging from 1 (not hungry/not thirsty) to 7 (very thirsty/very hungry). These data were entered into repeated measures ANOVAs with the factor *Time* (pre vs. post vs. end). Thirst ratings confirmed the results obtained for the trial-by-trial thirst ratings and are not reported for brevity.

Where appropriate, the Greenhouse-Geisser procedure was used to correct for violations of sphericity. *Post hoc* tests were conducted using Bonferroni correction.

### MRI Data Acquisition and Analysis

MR acquisition took place on a 1.5T Philips Intera MR system (Philips, Hamburg, Germany). For functional scanning, a T2* weighted Fast Field Echo, Echo Planar Imaging sequence utilizing parallel scanning technique was used (SENSE; Pruessmann et al., 1999). In plane resolution was 3 × 3 mm, and slice thickness was 3.5 mm (32 axial slices; no gap; FOV = 240 mm; acquisition matrix = 80 × 80; TE = 40 ms; flip angle = 90°; TR = 2500 ms). In addition, a standard T1 weighted high resolution structural scan with 1 × 1 × 1 mm voxel resolution was obtained.

Preprocessing and statistical analysis of the functional data was conducted using SPM8 (Wellcome Department of Imaging Neuroscience, University College London, UK; Friston et al., 1994). Preprocessing steps included realignment and slice time correction for the functional images. No subject displayed head movements exceeding 3 mm or 3° on any axis, and thus data from all participants were included in further analysis. Images were normalized to the MNI EPI template and resampled at 3 × 3 × 3 mm voxel size. A Gaussian spatial kernel of full width at half maximum (FWHM) with an 8 mm radius was used to smooth the data.

At the subject-level, the onset of water administration in each trial was modeled as event using the canonical hemodynamic response function (two gamma functions). All trials were then sorted into five time bins consisting of 10 trials each (trial 1–10, trial 11–20, trial 21–30, trial 31–40, trial 41–50). These time bins were then entered as regressors of interest into the SPM design matrix, which consisted of 12 regressors overall: five regressors of interest, representing the onsets of water administration for each time bin, six regressors of no interest, representing the movement parameters obtained during realignment, and one regressor incorporating an overall intercept to the model. For group-level random-effects analysis, the estimated betas of the five regressors of interest from all subjects were then entered into a flexible factorial model, which included the factor *Time* (Bin1 vs. Bin2 vs. Bin3 vs. Bin4 vs. Bin5) and a subject factor. Contrasts were computed for linear decreases [2 1 0 −1 −2] and linear increases [−2 −1 0 1 2] of the BOLD signal between time bins. All reported contrasts resulted in SPM(t) maps. Activations of these contrasts were considered as meaningful if they reached a single-voxel inclusion threshold of *p* < 0.001 (uncorrected) and a cluster-level threshold of *p* < 0.05 (FWE-corrected for multiple comparisons; family-wise error). As we had specific a priori-expectations regarding the potential involvement of insular regions, we also conducted a small volume correction by using a region-of-interest (ROI) encompassing the bilateral insula. This anatomic ROI was obtained using the maximum probability tissue atlas from the OASIS-project^2^) as provided in SPM12 by Neuromorphometrics, Inc., Somerville, MA, USA. under academic subscription^3^.

Furthermore, additional models were computed in order to correlate the BOLD signal during each trial with the corresponding intra-trial thirst and pleasantness ratings. Therefore, single subject data were modeled using the canonical hemodynamic response function (two gamma functions) in one session incorporating 50 regressors of interest, each representing the onset of water administration of a single trial, as well as six regressors of no interest, representing the movement parameters obtained during realignment, and one regressor incorporating an overall intercept to the model. Percent signal changes (PSC) were extracted using rfxplot (Gläscher, 2009). Cluster-mean PSC extraction was performed for each activated region obtained during analysis of the initial model. The correlation analysis was performed using SPSS software.

## Results

### Self-Report Data

#### Thirst Ratings

**Figure 2:**
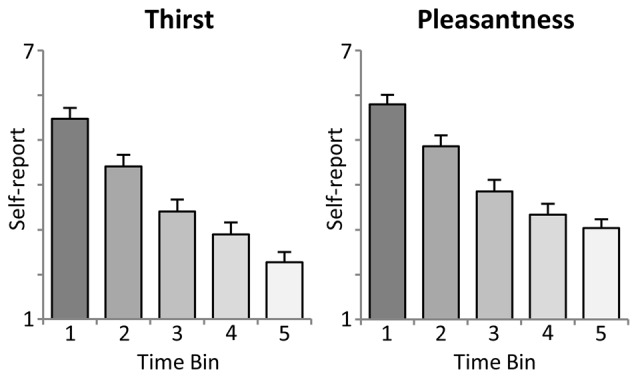
**Illustration of the intra-task thirst ratings (mean ± SEM)**. Each time bin represents an interval of 10 trials, i.e., the ingestion of 100 ml of water.

As shown in the upper panel of Figure 2, participants reported a significant linear decrease in thirst with increasing ingested water, i.e., after each time bin of 10 trials (First: *M* = 5.5, SD = 1.18; Second: *M* = 4.4, SD = 1.28; Third: *M* = 3.4, SD = 1.34; Fourth: *M* = 2.9, SD = 1.32; Fifth: *M* = 2.3, SD = 1.14). Statistical analysis revealed a significant linear trend, *F*_(1,23)_ = 163.5, *p* < 0.001, partial *η*^2^ = 0.88, and main effect of *Time*, *F*_(4,92)_ = 109.4, *p* < 0.001, partial *η*^2^ = 0.83.

#### Pleasure Ratings

Moreover, the analysis of water pleasantness revealed a highly significant main effect of *Time, F*_(4,92)_ = 78.7, *p* < 0.001, partial *η*^2^ = 0.77. As shown in the lower panel of Figure 2, pleasantness ratings decreased significantly from the first to the fourth time bin (First: *M* = 5.8, SD = 1.05; Second: *M* = 4.9, SD = 1.21; Third: *M* = 3.9, SD = 1.27; Fourth: *M* = 3.3, SD = 1.20), *ts*_(23)_ > 4.5, *p* < 0.001), but did not further decrease with the fifth time bin (*M* = 3.0, SD = 0.99), *t*_(23)_ = 2.2, *ns, linear trend: F*_(1,23)_ = 157.2, *p* < 0.001, partial *η*^2^ = 0.87.

#### Hunger Ratings

Participants were in neither a satiated nor deprived food state at the beginning of the experiment (Pre: *M* = 3.5, SD = 1.62), and there were no changes in reported hunger across the experiment (Post: *M* = 3.5, SD = 1.67; End: *M* = 3.8, SD = 1.89), *F*_(2,46)_ = 0.9, ns, partial *η*^2^ = 0.04.

### fMRI Data

**Figure 3:**
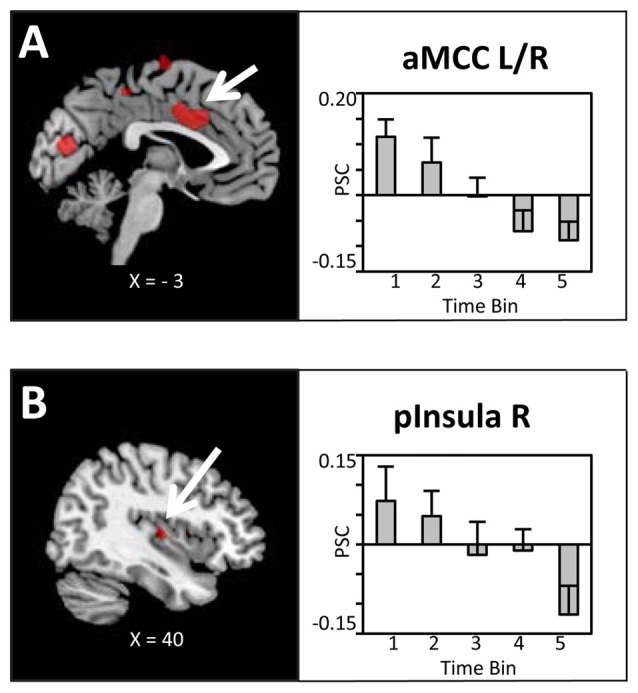
**Brain sections and extracted percent signal changes (PSC; mean ± SEM) illustrating the modulation in blood oxygenation level dependent (BOLD) activity in (A)** the anterior mid-cingulate cortex (aMCC) and **(B)** posterior insula proportional to the amount of water ingested. Statistical maps are thresholded with a cluster-defining voxel-level threshold of *p* < 0.001 (uncorrected) and a cluster-level threshold of *p* < 0.05 (FWE-corrected). Abbreviations: aMCC, anterior mid-cingulate cortex; pInsula, posterior insula.

The main hypothesis predicts a linear decrease in the BOLD signal which is proportional to the amount of water ingested. Thus, experimental trials were grouped into time bins (10 trials), each representing the ingestion of 100 ml of water, and a linear contrast of (*Bin1 > Bin2 > Bin3 > Bin4 > Bin5*) was computed. In line with our hypotheses, as illustrated in Figures 3A,B, linear decreases of the BOLD signal with increasing water consumption were observed in the anterior mid-cingulate cortex (aMCC) and, based on ROI analysis, the right posterior insula. Extracted PSC for these two regions showed a clear reduction in BOLD response distributed across the full period of water administration. Specifically, BOLD activity started with enhanced activation in response to water administration, gradually shifting towards deactivation of the BOLD response.

**Figure 4:**
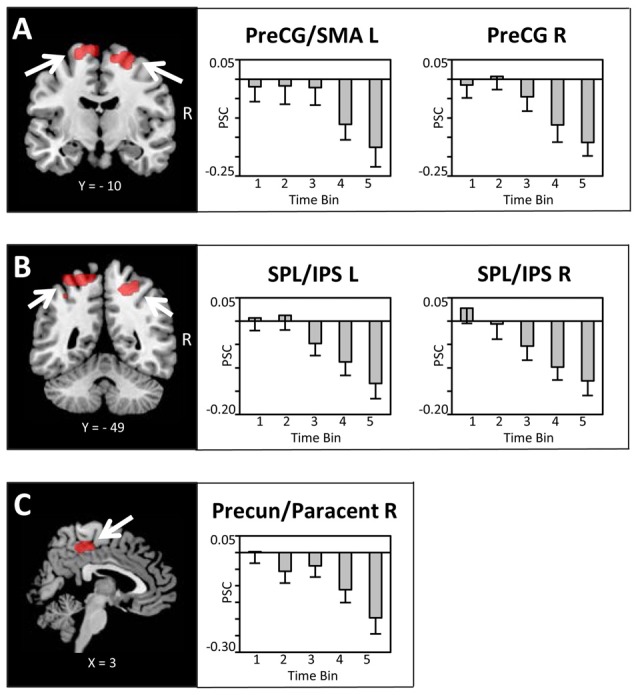
**Brain sections and extracted PSC (mean ± SEM) illustrating the modulation in BOLD activity in (A)** the supplementary motor cortex (SMA)/PreCG, **(B)** superior parietal lobule/intra-parietal sulcus (SPL/IPS) and **(C)** the Precun/Paracentr proportional to the amount of water ingested. Statistical maps are thresholded with a cluster-defining voxel-level threshold of *p* < 0.001 (uncorrected) and a cluster-level threshold of *p* < 0.05 (FWE-corrected). Abbreviations: SMA, supplementary motor cortex; PreCG, pre-central gyrus; SPL, superior parietal lobule; IPS, intra-parietal sulcus; Precun, precuneus; Paracentr, paracentral lobule.

Bilateral regions at/around the vicinity of the posterior aspect of the supplementary motor cortex (SMA; Figure 4A), bilateral superior parietal lobule/intra-parietal sulcus (SPL/IPS; Figure 4B), medial precuneus/paracentral lobule (PreCun/ParaCentr; Figure 4C), and left calcarine sulcus (Table 1) also showed a decrease in the BOLD signal with increasing amounts of fluid ingested. However, as these regions did not show enhanced activation to water stimuli in a deprived state (i.e., Bin 1), this reflects increased deactivation with increased water consumption. Finally, the left superior temporal gyrus (STG) and right middle temporal gyrus (MTG; Table 1) showed enhanced activation to water stimuli in Bin 1, which linearly decreased and did not switch towards deactivation.

**Table 1:** **Regions showing a modulation in blood oxygenation level dependent (BOLD) activity proportional to the amount of water ingested**.

		Coordinates					
Region	Side	*x*	*y*	*z*	# Voxels	*Z*	PSC × Thirst
Anterior middle cingulate cortex	L/R	−3	11	31	65	4.33	0.51***
Posterior insula	R	42	−10	7	9	3.41^a^	0.49***
Superior temporal gyrus/PT	L	−57	−31	13	82	4.34	0.52***
Middle temporal gyrus	R	57	−58	4	47	3.98	0.64***
Precentral gyrus/SFG	R	24	−10	64	66	4.75	0.57***
Precentral gyrus/SFG/SMA	L	−6	−7	73	117	4.32	0.53***
Superior parietal lobule/IPS	R	24	−40	64	125	4.33	0.70***
Superior parietal lobule/IPS	L	−12	−52	67	170	4.44	0.61***
Precuneus/paracentral lobule	L/R	3	−34	52	72	4.64	0.52***
Calcarine sulcus	L	−9	−79	16	108	5.58	0.62***

Cluster-defining voxel-level threshold p < 0.001 (uncorrected); cluster-level threshold p < 0.05 (FWE-corrected). *Side* indicates hemisphere in which the reported cluster peak voxel is located (R, right; L, left). *x/y/z* report MNI-coordinates. #Voxels reports cluster size in terms of voxel number. The rightmost column lists the single trial correlation between PSC and thirst (***p < 0.001). Abbreviations: IPS, intraparietal sulcus; PT, planum temporale; SFG, superior frontal gyrus; SMA, supplementary motor area; ^a^small volume corrected with bilateral insula-ROI.

The reverse contrast (*Bin1 < Bin2 < Bin 3 < Bin4 < Bin5*) did not reveal any significant activation, i.e., no brain region showed an increase in BOLD response proportional to the amount of water ingested.

### Correlation with Thirst and Pleasantness Ratings

**Figure 5:**
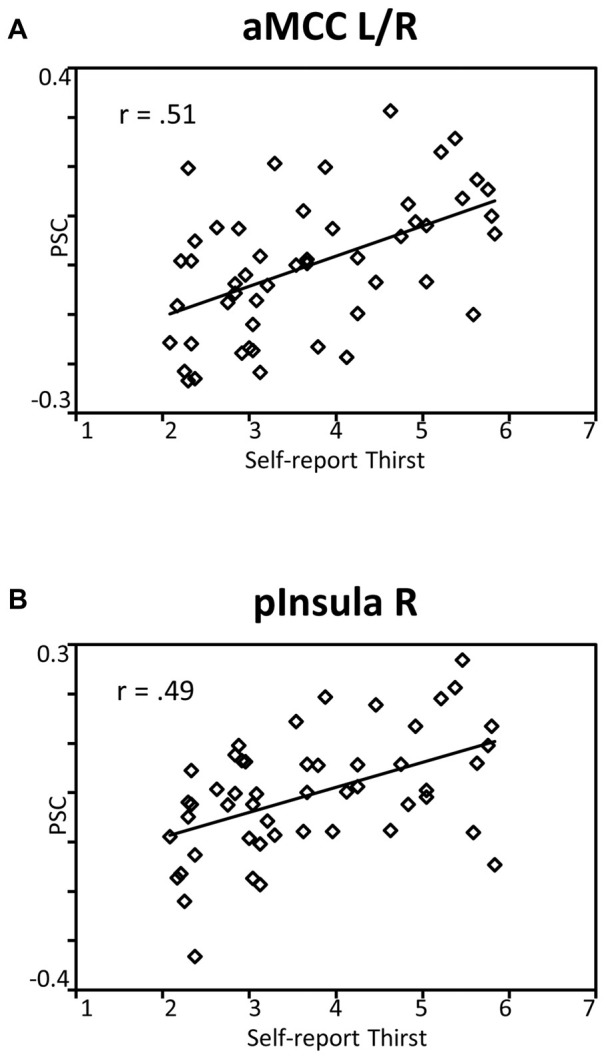
**Scatterplots showing the relationship between single trial PSC and corresponding thirst ratings in (A)** the aMCC and **(B)** posterior insula. Abbreviations: aMCC, anterior mid-cingulate cortex; pInsula, posterior insula.

To reveal the relationship between BOLD responses and participants’ thirst on a trial-by-trial basis, a correlation analysis with single trial PSC and corresponding thirst ratings was performed. Significant correlations between the BOLD signal and thirst ratings were detected for all regions modulated by water administration. Specifically, all correlations showed a positive relationship between PSC and thirst, i.e., a decrease of the moment-to-moment feeling of thirst was associated with a reduction in BOLD response to water ingestion, *r*’s > 0.49, *p*’s < 0.001 (see Table 1). With special focus on our* a priori* regions, this relationship is illustrated for the aMCC (Figure 5A) and posterior insula (Figure 5B). Furthermore, findings for the pleasure associated with drinking water were similar, which is expected given the high correlation of thirst and pleasantness ratings, *r* = 0.98, *p* < 0.001.

### Control Analysis

**Figure 6:**
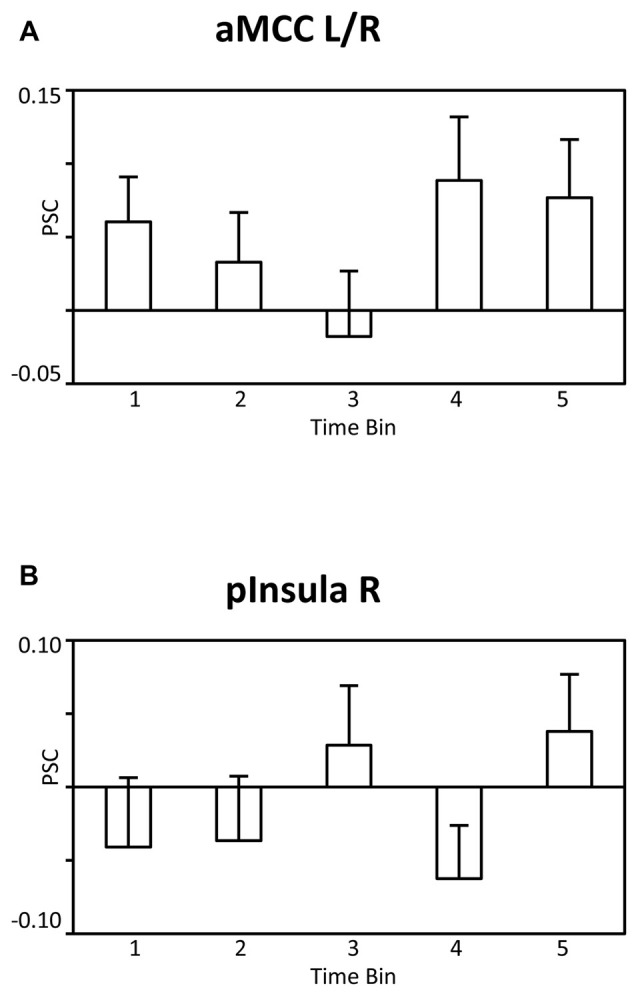
**Bar graphs showing the PSC in (A)** the aMCC and **(B)** posterior insula during the pleasantness rating period as a function of time bins. Abbreviations: aMCC, anterior mid-cingulate cortex; pInsula, posterior insula.

It is possible that the observed findings reflect unspecific effects across time, i.e., habituation or fatigue, rather than specific effects associated with satiety and the reduced incentive value of water. According to this reasoning, similar patterns of BOLD signal changes across time should be observed for the BOLD signal whether analysis is time-locked, as in the main analysis, or time-unlocked, i.e., in a period unrelated to water administration. To examine the issue, a control analysis was conducted in which the onset of the pleasantness rating period was analyzed identically to the main SPM model. As illustrated in Figures 6A,B for the aMCC and posterior insula, respectively, the BOLD responses were characterized by an unspecific pattern of BOLD signal changes rather than a linear decrease, linear trend: *F’s*_(1,23)_ = 2.4 and 0.6, *p*’s > 0.13. In sum, the findings strengthen the view that the linear decreases of the BOLD response in the aMCC and posterior insula relate to motivation effects rather than unspecific changes across time.

## Discussion

The sensation of thirst exerts a powerful effect on drinking. As everybody knows, the pleasure of drinking a glass of water is high when thirsty and low when satiated. This decrease of the incentive value of water over the course of drinking was used in the present study to identify neural structures representing dynamic changes in the motivation to drink associated with water consumption. Paralleling changes in incentive value, the functional imaging data revealed a decrease of the BOLD signal in the aMCC and posterior insula proportional to the amount of water administered. In addition, single trial BOLD responses of associated regions were related to trial-by-trial ratings of thirst and pleasantness. Overall, the aMCC and posterior insula appeared as key sites of a neural network for the motivation of drinking based on the dynamic integration of internal state and external stimuli.

Previous research on thirst and drinking primarily has contrasted discrete stages of deprivation and satiation. For instance, focusing on the anticipatory stage of behavior, a recent study examined the processing of need-related and control pictures during states of deprivation and satiety (Becker et al., 2015). The findings showed increased responses to beverage as compared to chair stimuli in the cingulate cortex, insular cortex, and the amygdala during the thirst state, which were absent in the no-thirst condition. Furthermore, examining the consummatory stage of behavior, previous research observed effects for the cingulate and insular cortices but not the amygdala. For instance, compared to no response during fluid satiation, a part of the mid-insular cortex showed a selectively increased BOLD response to water consumption when participants were in a need-state (de Araujo et al., 2003). This finding is further extended by a positive correlation between BOLD responses and pleasantness ratings in the aMCC, mid-insular cortex, and medial OFC (de Araujo et al., 2003). Similarly, increased drinking-related activation was also observed in the MCC and insular cortex in a recent study by Saker et al. (2014). Building upon these previous findings, the present study focused on dynamic, moment-to-moment changes in stimulus value representation. As shown in Figure 3, increasing water consumption led to a linear decrease in BOLD signal activation in the aMCC and posterior insula. Furthermore, the activity in the aMCC and posterior insula was significantly correlated with trial-by-trial changes in perceived thirst and pleasantness (see Figure 5). Both streams of analyses demonstrate gradual decreases of activation in the aMCC and posterior insula, appearing during early phases of fluid consumption and continuing over the course of the experiment. This pattern of findings suggests that the value representation of the water stimuli is adjusted according to cues associated with drinking, e.g., mouth sensation, swallowing and gastro-intestinal sensations, rather than the restoration of fluid balance (Rolls, 2005). In general, the adaptive organization of drinking behavior integrates information about the quality of the external stimulus and body state variables. Controlling for external stimulus quality by delivering the same water stimulus, the aMCC and posterior insula appeared as candidate neural structures for the representation of the value of water dynamically adjusted according to internal state.

The main finding was supported by two streams of analysis observing decreasing BOLD signal activity in the aMCC and posterior insula. However, the concern arises that the effect may reflect unspecific changes across time, such as habituation or fatigue. Although this hypothesis should presumably predict a representative pattern across widely distributed brain regions, a control analysis was undertaken to ensure that the effect is specific to the processing of water stimuli. Specifically, a different time period of the trial sequence was analyzed in the same way as the water stimulus-locked activity. Here, an unsystematic pattern of BOLD signal changes, clearly distinct to the linear decrease reported in the main analyses, was observed (see Figure 6). Thus, the control analysis strengthens the notion that the aMCC and insular cortex may be key sites for the dynamic modulation of external stimulus processing with internal body state.

Beyond thirst and drinking, the activation of the dorsal anterior cingulate cortex (dACC) has been revealed in a large number of studies examining other motivational processes such as eating, fear, and anxiety as well as emotional and self-relevant stimulus evaluation and reward processes (Yarkoni et al., 2011). Accordingly, the present findings may be considered from a broader perspective regarding the functional significance of the dACC. Heilbronner and Hayden (2016) recently sorted the extant literature on the dACC into interrelated categories of monitoring (error, conflict and reward monitoring), controlling (motor control, learning, self-control) and reward evaluation functions (e.g., action-outcome association). The organization of drinking behavior in natural environments places demands corresponding to each of these functions. Furthermore, a number of views discuss the dACC as a neural hub important to the sequential organization of extended goal-directed behaviors and the representation of contexts and moment-to-moment varying task-state variables (Etkin et al., 2011; Holroyd and Yeung, 2011; Shackman et al., 2011; Heilbronner and Hayden, 2016). The present findings are also consistent with this broader conception of dACC function, revealing that the dACC is involved in short-term adjustments of stimulus value in a specific survival-relevant motivational system.

The insular cortex, with its functional subdivisions, has been implicated in previous research in studies examining both thirst (Denton et al., 1999; Parsons et al., 2000; Egan et al., 2003; Farrell et al., 2006, 2008, 2011) and fluid consumption (de Araujo et al., 2003; Saker et al., 2014). Interestingly, de Araujo et al. (2003) found robust activations in the anterior insula/frontal operculum to water that was not modulated by thirst; instead, effects of body state were observed for the mid insular region. Similarly, Saker et al. (2014) observed satiation effects in the mid- and posterior insula. Corroborating these findings, the present study revealed a dynamic decrease in activation by water stimuli proportional to the amount of water in the right posterior insula. Interestingly, research on sensory specific satiety and overeating provides complementary evidence for an interaction of external stimuli and internal state in the insula. For instance, one study examined the processing of vanilla and banana odors in two sessions (pre- and post-meal) different only in that the second session occurred after eating bananas to satiety (O’Doherty et al., 2000). Similarly, a further study investigated changes in brain activity related to eating chocolate to satiety, tracking the hedonic experience of chocolate from being highly pleasurable to being highly aversive (Small et al., 2001). Both studies showed a decrease in activation, with satiation in regions of the insular cortex in the vicinity of the effects observed in the present study. Future studies determining common sites for eating and drinking regarding the interaction of internal state and external stimuli would be particularly informative with regard to conceptions of the insular cortex proposing functional subdivisions (Craig, 2009). Specifically, primarily based on the representation of pain, the posterior insula has been conceived as providing a representation of the physiological conditions of the body (Craig, 2009). According to the present findings, the posterior insula appears as region of information integration in that responses to water stimuli were modulated by fluid consumption.

Previous research observed effects associated with thirst and satiation in a number of cortical regions beyond the ACC and insular cortex. For instance, drinking-related activations have been observed in several brain regions during deprivation compared to satiety, including the SMA, IPL and STG (Saker et al., 2014). In the present study, the pattern of activation observed for the aMCC and insular cortex were paralleled in the SMA, SPL/IPS, PreCun/ParaCentr and calcarine sulcus, with the critical difference that most of these structures did not show an enhanced response to water stimuli when deprived. One may speculate that the BOLD deactivations found in the SMA, SPL/IPS, PreCun/ParaCentr and calcarine sulcus are associated with response inhibition and attentional modulation. For instance, the urge to drink had to be suppressed during the “hold” period at the beginning of the experiment, when participants were thirsty and drinking highly appreciated, and this conflict may have gradually faded with relief from thirst. This accords with research revealing that the SMA is associated with the inhibition of response plans and learning (Nachev et al., 2008), and the SPL/IPL involved in the control of attention and sensory-motor integration (Andersen and Buneo, 2002; Corbetta and Shulman, 2002; Shomstein, 2012). However, acknowledging the issue of reverse inference (Poldrack, 2006), systematic research is needed to examine these hypotheses.

Some limitations with regard to this study need to be considered. Compliance with the instruction was measured by means of self-report. While the data suggest that participants complied with the instruction to refrain from drinking for 7 h before the session, physiological measures of fluid deprivation would be preferable in future research. Furthermore, measures of swallowing would allow determining if participants were able to hold the water in the mouth. Preventing contamination of the functional imaging data by movement artifacts may be particularly relevant with regard to subcortical brain regions. A further limitation of the ability to detect small volume effects regards field strength (1.5 T) and signal acquisition. Thus, future studies utilizing improved fMRI parameters, including higher scanner field strength and optimized scanning sequences (e.g., pulsed arterial spin labeling; see Farrell et al., 2011), may reveal effects in small nuclei located in the hypothalamus, circumventricular organs, and interconnected brain regions implicated in the regulation of drinking behavior by animal research (Bourque, 2008; Leib et al., 2016).

## Conclusion

The power of external stimuli in the environment often varies with the current state of the organism. Thirst and drinking provides a useful model system for examining the neural correlates involved in representing state values. Paralleling a decrease in perceived pleasantness and thirst, the present findings showed that the activations elicited by water stimuli in the aMCC and posterior insula decreased in proportion to the amount of water consumed. These regions, which have been shown in previous research to be sensitive to discrete states of deprivation and satiation, also appear to provide a neural correlate for the motivation to drink based on the dynamic integration of external stimuli and internal state.

## Author Contributions

All listed authors contributed substantial work to the preparation of this study.

## Funding

This work was supported by the Federal Ministry of Education and Research within the projects EATMOTIVE (Grant 01EA1326, granted to BR and HS) and SmartAct (Grant 01EL1420A, granted to BR and HS) and the German Research Foundation (Re 3430, granted to BR).

## Conflict of Interest Statement

The authors declare that the research was conducted in the absence of any commercial or financial relationships that could be construed as a potential conflict of interest. The reviewer MA and handling Editor declared their shared affiliation, and the handling Editor states that the process nevertheless met the standards of a fair and objective review.

## Footnotes

^1^http://www.cedrus.com/lumina/

^2^http://www.oasis-brains.org/

^3^http://neuromorphometrics.com/
